# *PHACTR1* and *SLC22A3* gene polymorphisms are associated with reduced coronary artery disease risk in the male Chinese Han population

**DOI:** 10.18632/oncotarget.13506

**Published:** 2016-11-22

**Authors:** Qingbin Zhao, Huiyi Wei, Dandan Liu, Baolan Shi, Lei Li, Mengdan Yan, Xiyang Zhang, Fengjiao Wang, Yongri Ouyang

**Affiliations:** ^1^ Department of Geratology, The First Affiliated Hospital of Xi'an Jiaotong University, Xi'an, Shaanxi, 710061, China; ^2^ Department of Endocrinology, The First Affiliated Hospital of Xi'an Jiaotong University, Xi'an, Shaanxi, 710061, China; ^3^ Inner Mongolia Medical University, Hohhot, Inner Mongolia, 010050, China; ^4^ School of Life Science, Northwest University, Xi'an, Shaanxi, 710069, China; ^5^ Xi'an Tiangen Precision Medical Institute, Xi'an, Shaanxi 710075, China

**Keywords:** coronary artery disease (CAD), single nucleotide polymorphism (SNP), PHACTR1, SLC22A3, case-control study

## Abstract

Previous studies showed that *PHACTR1* and *SLC22A3* are involved in coronary vascular development and are key determinants of cardiovascular disease risk. We conducted a case-control study to examine the effect of *SLC22A3* and *PHACTR1* single nucleotide polymorphisms (SNPs) on CAD risk among 376 male CAD patients and 388 male healthy controls from China. Eleven *SLC22A3* and *PHACTR1* SNPs were selected and genotyped using Sequenom Mass-ARRAY technology. Odds ratios (OR) and 95% confidence intervals (CIs) were calculated using unconditional logistic regression adjusting for age. The rs9381439 minor allele “A” (OR = 0.72; 95% CI = 0.54–0.96; *p* = 0.024) in an allelic model was associated with reduced CAD risk, as were the rs2048327 “C/C” (OR = 0.60; 95% CI: 0.37–0.97; *p* = 0.036) and rs1810126 “T/T” (OR = 0.58; 95% CI: 0.36–0.93; *p* = 0.024) genotypes. Likewise, the rs9349379 “A/G” genotype in a dominant model (*p* = 0.041), the rs1810126 “T/C” genotype in additive (*p* = 0.041) and recessive (*p* = 0.012) models, and the rs2048327 “C/T” genotype in a recessive model were associated with decreased CAD risk (*p* = 0.016). These results suggest several *PHACTR1* and *SLC22A3* polymorphisms are associated with decreased CAD risk in the male Chinese Han population.

## INTRODUCTION

Coronary artery disease (CAD), including myocardial infarction, angina pectoris and arteriosclerosis of the coronary arteries, is a leading cause of illness, disability and death worldwide, particularly in older people [1–3]. In China, moreover, the prevalence of CAD is increasing. The annual mortality rate due to ischemic heart disease is currently 5% among males and 3.65% among females [4]. Several studies have identified gender differences in CAD susceptibility that likely account for the higher mortality rate among males [5, 6].

CAD is a multifactorial disease, and both acquired and inherited components are implicated in its etiology [7]. While many CAD risk factors can be ameliorated through lifestyle changes, such as diet and exercise, one's genetic make-up and family history of the disease are not modifiable [8, 9]. Understanding the genetic factors that contribute to the development of CAD continues to be a crucial element for improving methods of effective prevention, early diagnosis and therapeutic strategies.

Among the genetic factors thought to contribute to the risk of CAD are gene single-nucleotide polymorphisms (SNPs) [10, 11]. For example, SNPs in *PHACTR1* [12] and *SLC22A3* [13] are reported to have a strong association with CAD in Lebanon and Finland, respectively. *PHACTR1* encodes phosphatase and actin regulator 1, which can mediate increased oxidation of low-density lipoproteins and is required for vascular development, but may also mediate atherosclerosis in humans [14]. *SLC22A3* includes 11 exons and 10 introns and encodes organic cation transporter 3 (OCT3), which plays key roles in the inactivation of biogenic amines and the removal of toxic substances [15]. We selected 11 *PHACTR1* and *SLC22A3* SNPs and assessed their potential to affect CAD risk.

## RESULTS

A total of 376 patients with CAD and 388 healthy controls were enrolled in this case-control study. The demographic characteristics of the study population are shown in Table 1; note the significant difference in the age distribution between the case and control groups (*p* < 0.001).

**Table 1:** Characteristics of the patients and controls

Variables	Case	Control	Total	*p*
Sex				
Male	376	388	764	
Age, mean ±SD	59.60 ± 11.35	47.50 ± 10.64		< 0.001^a^

a *p* value was calculated using Welch's t tests.

Table 2 summarizes the minor allelic frequencies (MAFs) of the tested SNPs among the individuals in the case and control groups. All the SNPs in the control group were in Hardy-Weinberg equilibrium (HWE) (*p* > 0.05). The allelic frequencies of SNPs in the control group were similar to those of the HapMap Asian population. Using the χ ^2^ test, we found that rs9381439 located in *PHACTR1* was significantly associated with decreased CAD risk (OR = 0.72; 95% CI = 0.54–0.96; *p* = 0.024).

**Table 2:** Allele frequencies in patients and controls and odds ratios estimates for CAD

SNP ID	Genes	Band	Role	Alleles A^a^/B	HWE *p*	MAF		ORs	95 % CI	*p*
						Case	Control			
rs9381439	*PHACTR1*	6p24.1	Intron	A/G	0.852	0.274	0.291	0.72	0.54–0.96	0.024^*^
rs9349379	*PHACTR1*	6p24.1	Intron	A/G	0.609	0.122	0.163	0.92	0.74–1.16	0.498
rs4715166	*PHACTR1*	6p24.1	Intron	G/A	0.701	0.258	0.273	0.88	0.71–1.10	0.259
rs4715167	*PHACTR1*	6p24.1	Intron	T/C	0.906	0.283	0.309	0.89	0.71–1.11	0.289
rs4711939	*PHACTR1*	6p24.1	Intron	C/T	0.809	0.279	0.303	0.92	0.74–1.15	0.462
rs402219	*SLC22A3*	6q25.3	Intron	G/A	0.902	0.430	0.424	1.02	0.85–1.29	0.842
rs376563	*SLC22A3*	6q25.3	Intron	T/C	0.278	0.384	0.373	1.05	0.85–1.29	0.660
rs2174914	*SLC22A3*	6q25.3	Intron	C/G	0.525	0.396	0.399	0.99	0.80–1.21	0.906
rs2048327	*SLC22A3*	6q25.3	Intron	C/T	0.608	0.423	0.451	0.89	0.73–1.09	0.272
rs2457576	*SLC22A3*	6q25.3	Intron	C/G	0.397	0.399	0.404	0.98	0.80–1.20	0.844
rs1810126	*SLC22A3*	6q25.3	3′UTR	T/C	0.474	0.420	0.455	0.87	0.71–1.06	0.172

Abbreviations:

a Minor allele; SNP, single-nucleotide polymorphism; HWE, Hardy-Weinberg equilibrium; MAF, minor allelic frequency; OR, odds ratio; 95% CI: 95% onfidence interval.

* *p* < 0.05 indicates statistical significance.

The genotype frequencies of SNPs in the CAD and control groups showed that rs2048327 and rs1810126 were significantly associated with the risk of CAD (Table 3). The rs2048327 “C/C” genotype was associated with a reduced risk of CAD (OR=0.60; 95 % CI: 0.37– 0.97; *p* = 0.036), as was the rs1810126 “T/T” genotype (OR = 0.58; 95 % CI: 0.36–0.93; *p* = 0.024).

**Table 3:** Association between SNP genotypes and CAD risk in the male Chinese Han population

SNPs	Case			Control				Genotype model					
	AA	AB	BB	AA	AB	BB	BB-OR	AA-OR	95%CI	*p*	AB-OR	95%CI	*p*
rs9381439	7	78	291	9	108	270	1.00 (reference)	0.72	0.21–2.44	0.595	0.69	0.47–1.01	0.057
rs9349379	27	140	209	31	149	206	1.00	0.68	0.37–1.26	0.219	0.71	0.50–1.01	0.060
rs4715166	32	148	195	36	166	183	1.00	0.70	0.38–1.28	0.249	0.79	0.56–1.12	0.187
rs4715167	31	147	197	34	165	185	1.00	0.75	0.40–1.38	0.355	0.81	0.57–1.14	0.229
rs4711939	30	146	200	33	158	194	1.00	0.77	0.41–1.44	0.419	1.18	0.73–1.91	0.489
rs402219	74	175	127	68	190	126	1.00	1.18	0.73–1.91	0.489	0.86	0.59–1.24	0.421
rs376563	51	187	138	59	171	157	1.00	1.11	0.67–1.85	0.680	1.43	0.99–2.04	0.051
rs2174914	57	184	135	65	179	143	1.00	1.63	0.63–1.69	0.894	1.24	0.86–1.77	0.246
rs2048327	63	192	121	81	186	119	1.00	0.60	0.37–0.97	0.036^*^	1.01	0.69–1.46	0.979
rs2457576	57	186	133	67	177	141	1.00	1.00	0.61–1.64	0.989	1.27	0.89–1.83	0.190
rs1810126	62	192	122	84	185	119	1.00	0.58	0.36–0.93	0.024^*^	0.98	0.67–1.41	0.905

* *p* < 0.05 indicates statistical significance;

A/B stands for minor/major alleles.

**Table 4:** Logistic regression analysis of the association between SNPs and CAD risk

SNP	Minor allele	Dominant model		Additive model		Recessive mode	
		OR (95% CI)^a^	*p*	OR (95% CI)^a^	*p*	OR (95% CI)^a^	*p*
rs9381439	A	0.69(0.48–1.00)	0.052	0.73(0.52–1.02)	0.064	0.79(0.23–2.67)	0.701
rs9349379	A	0.71(0.51–0.99)	0.041^*^	0.78(0.60–1.00)	0.053	0.78(0.43–1.42)	0.415
rs4715166	G	0.78(0.56–1.08)	0.129	0.82(0.64–1.06)	0.123	0.78(0.44–1.39)	0.398
rs4715167	T	0.79(0.57–1.11)	0.179	0.84(0.65–1.19)	0.185	0.82(0.45–1.49)	0.518
rs4711939	C	0.82(0.59–1.14)	0.234	0.86(0.66–1.11)	0.240	0.84(0.46–1.54)	0.567
rs402219	G	0.93(0.66–1.32)	0.715	1.05(0.83–1.32)	0.685	1.29(0.85–1.98)	0.235
rs376563	T	1.35(0.96–1.89)	0.083	1,14(0.89–1.44)	0.296	0.91(0.57–1.46)	0.709
rs2174914	C	1.18(0.84–1.66)	0.332	1.06(0.84–1.34)	0.638	0.92(0.59–1.43)	0.701
rs2048327	C	0.87(0.61–1.24)	0.443	0.80(0.63–1.01)	0.062	0.59(0.39–0.91)	0.016*
rs2457576	C	1.19(0.85–1.69)	0.298	1.05(0.83–1.33)	0.686	0.87(0.56–1.36)	0.552
rs1810126	T	0.84(0.59–1.19)	0.343	0.78(0.62–0.99)	0.041^*^	0.59(0.39–0.89)	0.012^*^

* *p* < 0.05 indicates statistical significance;

a OR (95% CI) and *p*-values were adjusted for age

The minor allele of each SNP was assumed to be a risk allele compared to the wild-type allele. Three models – additive, dominant and recessive models – were applied to analyze the association between SNPs and CAD after adjusting for age. We found that *PHACTR1* rs9349379 was associated with reduced CAD risk in a recessive model (OR = 0.71; 95% CI = 0.51–0.99; *p* = 0.041). In addition, *SLC22A3* rs1810126 was significantly associated with decreased CAD risk in an additive model (OR = 0.78; 95% CI = 0.62–0.99; *p* = 0.041) and recessive model (OR = 0.59; 95%CI = 0.39-0.89; *p* = 0.012). *SLC22A3* rs2048327 was also associated with reduced CAD risk in a recessive model (OR = 0.59; 95% CI = 0.39–0.91; *p* = 0.016). No other polymorphisms tested were associated with CAD risk in the Chinese Han male population.

## DISCUSSION

In this study we investigated the associations between the 11 SNPs in *SLC22A3* and *PHACTR1* and CAD risk in the Chinese Han male population. Among these, *PHACTR1* rs9381439 and rs9349379 and *SLC22A3* rs2048327 and rs1810126 contributed to a decreased risk of CAD in individual SNP analyses. These results suggest that *PHACTR1* and *SLC22A3* polymorphisms may be important determinants of the risk of CAD in the Chinese Han male population.

*PHACTR1* is a regulator of protein phosphatase 1 (PP1), an enzyme involved in regulating endothelial production of nitric oxide [16], an important modulator of cardiovascular homeostasis whose activity is elevated in in patients with end stage heart failure [17, 18]. In a genome-wide association study, *PHACTR1* rs9349379 was associated with genetic risk of CAD [19, 20]. Rs9349379 is situated within an intronic region of *PHACTR1* and is not in high linkage disequilibrium with any coding SNPs. It may therefore affect transcriptional regulation of *PHACTR1* [20]. In our study, we demonstrated that *PHACTR1* rs9349379 polymorphism exerts a potentially protective effect against CAD in the Chinese Han male population, which is consistent with an observation made in Lebanese cohort [12]. It could be supposed that the effect of *PHACTR1* rs9349379 polymorphism on CAD is not a single-factor process, but consists of complicated elements. We also showed that *PHACTR1* rs9381439 is associated with a 0.72-fold risk of CAD. To the best of our knowledge, this is the first report that this SNP is associated with CAD, though it was a relatively small-scale study, and further studies will be needed to confirm these results.

*SLC22A3* is located on 6p25.3 and is a member of the solute carrier super family. It encodes organic cation transporter 3 (OCT3) [21], which modulates intracellular histamine levels by mediating its release and uptake [22]. Histamine is a strong inflammatory mediator that can increase the permeability of vascular endothelial cells and thus increase the deposition of low-density lipoprotein (LDL) within vascular endothelial cells, thereby promoting formation of atherosclerotic plaques [23]. Histamine also induces expression of cytokines, chemokines and adhesion molecules and other inflammatory factors that contribute to atherosclerotic plaque formation [24]. Rs2048327 and rs1810126 reportedly downregulate *SLC22A3* transcription and OCT3 protein levels [25]. Individuals carrying these variants may therefore have altered histamine levels, which could greatly affect morbidity. It is also plausible that, by themselves, these polymorphisms have on impact on CAD risk.

Although the present study possesses sufficient power, some limitations should be considered. As we all know, CAD is very heterogeneous disease and has numerous risk factors, including poor nutrition, obesity, hypertension and diabetes. Because the sample size in our study was relatively small, and detailed information was lacking, we could not explore how genetic polymorphisms interact with those factors in CAD. The gene-gene and gene-environment interactions of *PHACTR1* and *SLC22A3* will need to be evaluated in future studies.

In sum, our study suggests that several *PHACTR1* and *SLC22A3* gene polymorphisms may exert a protective effect against the CAD in the Chinese Han male population. However, the precise function of these polymorphisms and the mechanisms by which expression of these genes affect CAD risk have yet to be determined.

## MATERIALS AND METHODS

### Study participants

All participants in our study were male Han Chinese. Between 2014 and 2016, we recruited a total of 376 patients diagnosed with CAD at the First Affiliated Hospital of Xi'an Jiaotong University and 388 healthy controls. All cases were verified, and the patients were recruited without restriction regarding age or disease stage and were previously in good health. Diagnosis was based on standard coronary angiography, which revealed ≥ 70% stenosis of the main branch of a coronary artery or aortic stenosis ≥ 50%). The patients had no history of cancer, infection, nephropathy or autoimmune disease. Patients with a history of myocardial infarction, stable angina or unstable angina were also classified as CAD subjects. Excluded were subjects with chronic diseases or conditions involving the brain, liver, heart or lung, and patients with more advanced cardiovascular, metabolic or endocrine diseases. Controls were unrelated and were selected randomly. All of the controls were in good health. All were required to have a comprehensive physical examination. None had any kind of cancer, heart disease or endocrinological, metabolic or nutritional disease.

### Clinical data and demographic

After each subject gave written informed consent, they were interviewed by a psychiatrist who collected personal information, which included region of residence, age, education status and family history of cancer. The use of samples was approved by the Human Research Committee of the First Affiliated Hospital of Xi'an Jiaotong University for Approval of Research Involving Human Subjects.

### SNP selection and genotyping

Among the 11 SNPs selected, rs2048327 in *SLC22A3* [13] and rs9349379 in *PHACTR1* [26] are reportedly associated with CAD; other *PHACTR1* and *SLC22A3* gene SNPs were chosen at random [12, 13]. Minor allele frequencies of all SNPs were >5% in the HapMap of the Chinese Han Beijing population. We used a GoldMag-Mini Whole Blood Genomic DNA Purification Kit (GoldMag Co. Ltd. Xi'an City, China) to extract DNA from whole blood. Extracted DNA was quantified using NanoDrop2000. Genotyping was done with the Sequenom MassARRAY RS1000 system using the standard protocol recommended by the manufacturer. The multiplexed SNP MassEXTENDED assay was designed using Sequenom Mass-ARRAY Assay Design 3.0 Software [27]. Data were managed and analyzed using Sequenom Typer 4.0 Software. Genotype was analyzed using a Sequenom MassARRAY RS1000 system and the standard protocol recommended by the manufacturer [27, 28].

### Statistical analysis

Statistical analyses were done using Microsoft Excel and SPSS 18.0 (SPSS, Chicago, IL, USA). All *p* values were two-sided, and *p* ≤ 0.05 was considered significant. Control genotype frequencies for each SNP were tested for departure from the HWE using Fisher's exact test. The χ^2^ test was used to compare the distribution of marker alleles and genotypes in the patients and controls [29]. Unconditional logistic regression analysis, adjusted for age, was used to test odds ratios (ORs) and 95% confidence intervals (CIs) [30]. Associations between SNPs and risk of CAD were tested in genetic models using SNP Stats software (http://bioinfo.iconcologia.net). Values of OR and 95% CI were calculated as above. Finally, SHEsis software (http://analysis.bio-x.cn/myAnalysis.php) was used to estimate pairwise linkage disequilibrium (LD) and haplotype construction [31].

## References

[1] Gaziano TA (2005). Cardiovascular disease in the developing world and its cost-effective management. Circulation.

[2] Lopez AD, Mathers CD, Ezzati M, Jamison DT, Murray CJ (2006). Global and regional burden of disease and risk factors, 2001: systematic analysis of population health data. Lancet.

[3] Gaziano TA (2007). Reducing the growing burden of cardiovascular disease in the developing world. Health Affair.

[4] Wang Q (2005). Molecular genetics of coronary artery disease. Curr Opin Cardiol.

[5] Residori L, Garcia-Lorda P, Flancbaum L, Pi-Sunyer FX, Laferrere B (2003). Prevalence of co-morbidities in obese patients before bariatric surgery: effect of race. Obes Surg.

[6] Kazemi-Saleh D, Pishgoo B, Farrokhi F, Fotros A, Assari S (2008). Sexual function and psychological status among males and females with ischemic heart disease. J Sex Med.

[7] Libby P, Theroux P (2005). Pathophysiology of coronary artery disease. Circulation.

[8] Bruneau BG (2008). The developmental genetics of congenital heart disease. Nature.

[9] Yusuf S, Hawken S, Ounpuu S, Dans T, Avezum A, Lanas F, McQueen M, Budaj A, Pais P, Varigos J, Lisheng L, Investigators IS (2004). Effect of potentially modifiable risk factors associated with myocardial infarction in 52 countries (the INTERHEART study): case-control study. Lancet.

[10] Ronald J, Rajagopalan R, Cerrato F, Nord AS, Hatsukami T, Kohler T, Marcovina S, Heagerty P, Jarvik GP (2011). Genetic variation in LPAL2, LPA, and PLG predicts plasma lipoprotein(a) level and carotid artery disease risk. Stroke.

[11] Boes E, Coassin S, Kollerits B, Heid IM, Kronenberg F (2009). Genetic-epidemiological evidence on genes associated with HDL cholesterol levels: a systematic in-depth review. Exp Gerontol.

[12] Hager J, Kamatani Y, Cazier JB, Youhanna S, Ghassibe-Sabbagh M, Platt DE, Abchee AB, Romanos J, Khazen G, Othman R, Badro DA, Haber M, Salloum AK (2012). Genome-wide association study in a Lebanese cohort confirms PHACTR1 as a major determinant of coronary artery stenosis. PloS one.

[13] Sallinen R, Kaunisto MA, Forsblom C, Thomas M, Fagerudd J, Pettersson-Fernholm K, Groop PH, Wessman M, Finnish Diabetic Nephropathy Study G (2010). Association of the SLC22A1, SLC22A2, and SLC22A3 genes encoding organic cation transporters with diabetic nephropathy and hypertension. Ann Med.

[14] Reschen ME, Gaulton KJ, Lin D, Soilleux EJ, Morris AJ, Smyth SS, O'Callaghan CA (2015). Lipid-induced epigenomic changes in human macrophages identify a coronary artery disease-associated variant that regulates PPAP2B Expression through Altered C/EBP-beta binding. PLoS Genet.

[15] Sakata T, Anzai N, Kimura T, Miura D, Fukutomi T, Takeda M, Sakurai H, Endou H (2010). Functional analysis of human organic cation transporter OCT3 (SLC22A3) polymorphisms. J Pharmacol Sci.

[16] Mount PF, Kemp BE, Power DA (2007). Regulation of endothelial and myocardial NO synthesis by multi-site eNOS phosphorylation. J Mol Cell Cardiol.

[17] Dudzinski DM, Michel T (2007). Life history of eNOS: partners and pathways. Cardiovasc Res.

[18] Neumann J, Eschenhagen T, Jones LR, Linck B, Schmitz W, Scholz H, Zimmermann N (1997). Increased expression of cardiac phosphatases in patients with end-stage heart failure. J Mol Cell Cardiol.

[19] O'Donnell CJ, Kavousi M, Smith AV, Kardia SL, Feitosa MF, Hwang SJ, Sun YV, Province MA, Aspelund T, Dehghan A, Hoffmann U, Bielak LF, Zhang Q (2011). Genome-wide association study for coronary artery calcification with follow-up in myocardial infarction. Circulation.

[20] Coronary Artery Disease Genetics C (2011). A genome-wide association study in Europeans and South Asians identifies five new loci for coronary artery disease. Nat Genet.

[21] Verhaagh S, Schweifer N, Barlow DP, Zwart R (1999). Cloning of the mouse and human solute carrier 22a3 (Slc22a3/SLC22A3) identifies a conserved cluster of three organic cation transporters on mouse chromosome 17 and human 6q26-q27. Genomics.

[22] Schneider E, Machavoine F, Pleau JM, Bertron AF, Thurmond RL, Ohtsu H, Watanabe T, Schinkel AH, Dy M (2005). Organic cation transporter 3 modulates murine basophil functions by controlling intracellular histamine levels. The J Exp Med.

[23] Rozenberg I, Sluka SH, Rohrer L, Hofmann J, Becher B, Akhmedov A, Soliz J, Mocharla P, Boren J, Johansen P, Steffel J, Watanabe T, Luscher TF (2010). Histamine H1 receptor promotes atherosclerotic lesion formation by increasing vascular permeability for low-density lipoproteins. Arterioscl Throm Vas.

[24] Kimura S, Wang KY, Tanimoto A, Murata Y, Nakashima Y, Sasaguri Y (2004). Acute inflammatory reactions caused by histamine via monocytes/macrophages chronically participate in the initiation and progression of atherosclerosis. Pathol Int.

[25] Nies AT, Koepsell H, Winter S, Burk O, Klein K, Kerb R, Zanger UM, Keppler D, Schwab M, Schaeffeler E (2009). Expression of organic cation transporters OCT1 (SLC22A1) and OCT3 (SLC22A3) is affected by genetic factors and cholestasis in human liver. Hepatology.

[26] Reschen ME, Lin D, Chalisey A, Soilleux EJ, O'Callaghan CA (2016). Genetic and environmental risk factors for atherosclerosis regulate transcription of phosphatase and actin regulating gene PHACTR1. Atherosclerosis.

[27] Gabriel S, Ziaugra L, Tabbaa D, Haines Jonathan L (2009). SNP genotyping using the Sequenom MassARRAY iPLEX platform. Current protocols in human genetics.

[28] Thomas RK, Baker AC, Debiasi RM, Winckler W, Laframboise T, Lin WM, Wang M, Feng W, Zander T, MacConaill L, Lee JC, Nicoletti R, Hatton C (2007). High-throughput oncogene mutation profiling in human cancer. Nat Genet.

[29] Adamec C (1964). [Example of the Use of the Nonparametric Test. Test X2 for Comparison of 2 Independent Examples]. Ceskoslovenske zdravotnictvi.

[30] Bland JM, Altman DG (2000). Statistics notes. The odds ratio. Bmj.

[31] Shi YY, He L (2005). SHEsis, a powerful software platform for analyses of linkage disequilibrium, haplotype construction, and genetic association at polymorphism loci. Cell Res.

